# Environmentally safe release of plant available potassium and micronutrients from organically amended rock mineral powder

**DOI:** 10.1007/s10653-020-00677-1

**Published:** 2020-08-25

**Authors:** B. B. Basak, Binoy Sarkar, Ravi Naidu

**Affiliations:** 1 ICAR-Directorate of Medicinal and Aromatic Plants Research, Anand, Gujarat 387310 India; 2 Lancaster Environment Centre, Lancaster University, Lancaster, LA1 4YQ UK; 3 Global Centre for Environmental Remediation, The University of Newcastle, Callaghan, NSW 2308 Australia; 4 Cooperative Research Centre for Contamination Assessment and Remediation of the Environment, ATC Building, Callaghan, NSW 2308 Australia

**Keywords:** Nutrient recycling, Rock dust, Organic matter, Potassium, Plant micronutrients, Potentially toxic elements

## Abstract

The staggering production of rock dusts and quarry by-products of mining activities poses an immense environmental burden that warrants research for value-added recycling of these rock mineral powders (RMP). In this study, an incubation experiment was conducted to determine potassium (K) and micronutrients (Zn, Cu, Fe and Mn) release from a quarry RMP to support plant nutrition. Four different size fractions of the RMP were incubated with organic amendments (cow dung and legume straw) under controlled conditions for 90 days. Samples were collected at different intervals (7, 15, 30, 45, 60 and 90 days) for the analysis of available K and micronutrients in the mineral-OM mixtures and leachates. There was a significant (*p *<0.05) increase in pH of leachates from the mineral-OM mixtures. The K release was significantly higher from the finer size fraction of RMP. About 18.7% Zn added as RMP was released during the incubation period. Zn release increased from 4.7 to 23.2% as the particle size of RMP decreased. Similarly, Cu release from RMP increased from 2.9 to 21.6%, with a decrease in the particle size. Fe and Mn recovery from RMP recorded 11.2 and 6.6%, respectively. Combined application of OM and RMP showed significantly higher nutrient release than other treatments. This study indicates that effective blending of RMP with organic amendments could be a potential source of K and micronutrients in agriculture without posing a risk of toxic element contamination to the soil.

## Introduction

Limited supply of potassium (K) and micronutrients (e.g., Zn, Cu, Fe, Mn) is the most prevalent constrain for plant growth worldwide, especially in organic agriculture where the nutrient supply to crops is mainly derived from mineralization/decomposition of native and/or introduced organic sources (e.g., manures and crop residues) and natural weathering of soil minerals (Torma et al. 2018). In conventional agriculture, micronutrients are supplied through commercial fertilizers, which are not permitted in organic agriculture (Codex Alimentarius Commission 2007). The nutrient requirements in organic agriculture may be externally met by the application of less soluble sources such as manures, crop residues and rock mineral powders (RMP) (Basak and Sarkar 2017). Presently, the main objective in the agricultural reforms worldwide is to promote a system that supports environmental sustainability and quality production, and organic agriculture is a promising practice to achieve this objective (COM 2004). Since synthetic sources are not allowed in organic cultivation system, the demand of nutrients is to be met by alternative sources such as organic materials and locally available geological materials (also termed as agro-minerals) (Manning 2018). It is imperative to find alternative sources that meet the guidelines of organic cultivation systems.

There are few but consistent reports on the use of multi-nutrient rock and mineral fertilizers in the organic and conventional production systems (Fyfe et al. 2006). RMP have recently been reported as a source of plant nutrients in Australia (Bolland and Baker 2000), Germany (von Wilpert and Lukes 2003), Brazil (Leonardos et al. 2000; Theodoro and Leonardos 2006; Ramos et al. 2017; Dalmora et al. 2020a), United Kingdom (Mohammed et al. 2014), and India (Basak 2019).

Organic farming encourages the application of natural and environmentally friendly alternative nutrient sources alone or in combination to improve their individual effectiveness. The application of RMP blended with composts was found as much as doubly effective in improving crop yields compared to RMP alone (Theodoro and Leonardos 2006). The combined application of RMP with organics (e.g., manure and compost) can be a promising approach because insoluble nutrients in RMP can transform into bioavailable forms by the action of organic acids produced during organic matter decomposition (Basak et al. 2017). The organic acids promote mineral dissolution directly through the supply of protons which attack the alumino-silicate structure of the rock. The conjugate base is also responsible for forming a complex with metal ions (Al^3+^, Fe^2+^ and Si^4+^) in the alumino-silicate framework, and subsequently releases nutrients in the solution (Song et al. 2015). The positive effect of organic acids on mineral dissolution thus can be defined as the sum of the proton-promoted (*H*) and ligand-promoted (*L*) effects (Eq. 1) (Ullman and Welch 2002):

1 $$R_{t} = RH + RL = k_{1} aH^{m} + k_{2} aL^{n}$$

where, *R*_*t*_ is the release of ions at time *t; m* and *n* are empirical exponent constants; *k*_1_ and *k*_2_ are kinetic rate constants for proton- and ligand-promoted dissolution; and *aH* and *aL* are activities of proton- and ligand-promoted ion species.

The dissolution rate of mineral can further increase by the combined effects of organic acids and CO_2_ produced within the soil (e.g., soil respiration, organic matter decomposition) or drawn down from the atmosphere because carbonic acid participates directly in the silicate weathering reaction (Beerling et al. 2018). Following these reactions, plant micronutrients such as Cu, Zn, Fe and Mn can become bioavailable in neutral to alkaline soil solutions in the form of organic complexes (Rengel 2015).

Approximately 7–17 billion tons of rock dusts and quarry by-products are generated annually as a result of mining activities globally including in Australia, Africa and Latin America (Renforth et al. 2011). The mining by-products can be an environmental liability if left stagnating at the site for long time. RMP obtained as mining by-products are silicate minerals composed mainly of quartz, plagioclase, mica and feldspar, but may vary considerably in their composition (Manning 2018). RMP can serve as the source of multiple plant-nutrients when applied to soils (van Straaten 2002). These practices would not only provide a low-cost source of plant nutrients to farmers (Codex Alimentarius Commission 2007), but also would find a value-added option for sustainable quarry by-product disposal. However, a number of factors are needed to be considered for selecting RMP in agriculture such as (1) at what space RMP supplies nutrients to plants and does it meet the crop demand, (2) how the particle size of RMP influences its nutrient release, and (3) how organic amendments influence the nutrient release rates. Very little information is available in the literature on the nutrient supplying potential, specially K and micronutrients of RMP from an agricultural point of view.

To this end, here we evaluated a RMP collected from a quarry located in New South Wales (NSW), Australia, as a source of K and micronutrients through various laboratory incubation experiments, and also attempted to understand the implication of RMP utilization in the plant-soil nutrient management system. We hypothesized that mineral dissolution will happen during the incubation of RMP with organic matter due to the production of organic acids and CO_2_ (Basak et al. 2017), and the nutrient solubilization (bioavailability) will be facilitated by low molecular weight organic acids produced during organic matter decomposition (Badr 2006).

## Materials and methods

### Rock mineral powder (RMP)

The RMP was collected from the crushed by-product of a mining quarry located at Seaham, New South Wales, Australia (32.799′′S 151.635°E). The collected samples were ground in a steel grinder. The RMP sample was distributed into four size fractions of particles using a mechanical sieve shaker: (1) 16 mesh sieve (passed through 16-mesh sieve, but retained on 18-mesh sieve), (2) 30 mesh sieve (passed through 30-mesh sieve, but retained on 36-mesh sieve), (3) 60 mesh sieve (passed through 60-mesh sieve, but retained on 72-mesh sieve), and (4) 120 mesh sieve (passed through 120-mesh sieve, but retained on 150-mesh sieve). The corresponding particle sizes of the four different fractions were 1000, 500, 250 and 125 µm, respectively.

### Organic materials and quartz

A well-decomposed cow dung and a legume (*Pisum sativum*) straw were purchased from a local garden store (Bunnings Warehouse, Newcastle, Australia). The cow dung and legume straw were oven dried at 35 °C for 96 h, ground with a Wiley mill, and sieved through a 0.5-mm sieve for further use. Guaranteed reagent (GR) grade quartz (SiO_2_) particles (Merck, USA) were used as an inert medium for the laboratory incubation study.

### Total elemental analysis

The total elemental composition in percentage weight of oxides in the RMP sample (125 µm) was determined by X-ray Fluorescence Spectroscopy (Axios MAX, PANalytical, Netherlands) using standard-less software (Omnian software). Total K, micronutrients (Zn, Cu, Fe and Mn) and potentially toxic element (PTE) contents in the RMP were analyzed by microwave assisted digestion of the material in aqua regia (EPA 1996, Method 3050B). The RMP sample (0.1 g) was weighted in a Teflon tube, treated with 10 mL of aqua regia. The sample was placed in the microwave digestion system (CEM5000, CEM Corporation, USA) for digestion (750 W at 500 k Pa for 30 min). Similarly, the cow dung and legume straw were digested in concentrated pure nitric acid (HNO_3_). Each digested sample was filtered through a 0.45-μm nylon filter, and elements of interest were analyzed using an inductively coupled plasma-mass spectrophotometer (ICP-MS) (Agilent 7700 × ICP-MS, Agilent Technologies Inc., USA). The ICP-MS was equipped with a concentric nebulizer 7500 CX quadrupole mass analyzer and ORS collision chamber. Helium (5.5 purity) was used at an equal flow rate for all elements. Prior to measurements, the ICP-MS was calibrated using a multi-elemental standard (ICP-AM-3 high priority standards, USA). Nitric acid (2%) blanks and mid-level standards were run after every ten samples for quality control.

### Incubation study

An incubation experiment was conducted to determine K and micronutrients (Zn, Cu, Fe and Mn) release from the RMP collected from the quarry. The different size fractions of the RMP were incubated under controlled conditions with organic amendments. Finely ground legume straw (LS) and cow dung (CD) were used as the organic amendments. The fine granular quartz sand was washed and used as inert medium for the laboratory incubation studies. Six treatments consisting of four size fractions of the RMP and organic materials are as follows:

- *T*_1_: Control (quartz sand)
- *T*_2_: Quartz + LS + CD
- *T*_3_: Quartz + LS + CD + RMP (2000 µm)
- *T*_4_: Quartz + LS + CD + RMP (500 µm)
- *T*_5_: Quartz + LS + CD + RMP (250 µm)
- *T*_6_: Quartz + LS + CD + RMP (150 µm)

The experiment was laid out in a completely randomized block design with three replications for each treatment. Quartz was used as a base material in all the treatments. The quartz particles (size 0.2–0.5 mm) were washed with dilute HCl (0.15%) twice to remove any unwanted materials, and rinsed twice with distilled water. For the incubation study, air-dried 250 g quartz was mixed and homogenized with 2.5 g of LS, 2.5 g of RMP and 25 g of CD. The study materials were moistened to an optimum level (60% of water holding capacity) and incubated at 25 °C for 90 days in dark. Citric acid is the most common organic acid present in the rhizosphere of majority of plants (Marschner 1995), and it was used for the incubation studies to simulate the chemical environment similar to the rhizosphere. Each treatment was mixed with 150 mL of citric acid (0.01 M) solution at the start of the incubation.

The mixtures were then placed on a plastic funnel lined with Whatman No 1 filter paper, and fixed on a 500-mL capacity conical flask. The moisture contents of the mixture were kept constant by weighing the sample every alternative day and adding required volume of double distilled water. Then, the mixture was incubated for different periods (7, 15, 30, 45, 60 and 90 days) at 25 ± 0.5 °C. Samples were collected at different intervals (7, 15, 30, 45, 60 and 90 days) for the analysis of available K and micronutrients. Samples were leached frequently (once in 15 days) with distilled water to analyze total soluble K and micronutrients fallowing collection of the filtrate through a suction pump. After filtration (Whatman No. 42 filter paper), K content in the leachate was estimated on a flame photometer (FP 6420, MesuLab, China) following suitable dilution. A quality control set consisting of a calibration blank and a mid-level calibration standard were run every ten samples. The rate of K release from RMP was computed by fitting the data obtained from leaching experiments into the first-order kinetic equation (Jardine and Sparks 1984) (Eq. 2):

2 $$\ln \left( {a{-}p} \right) = \ln a{-}kt$$

where *a* = amount of K release initially; *p* = amount of K release at a particular time ‘*t*’; (*a* − *p*) = amount of K present finally, and *k* = rate constant. The kinetic data fitting was evaluated based on the values of correlation coefficient (*r*) and standard error of estimate (SE).

**Fig. 1:**
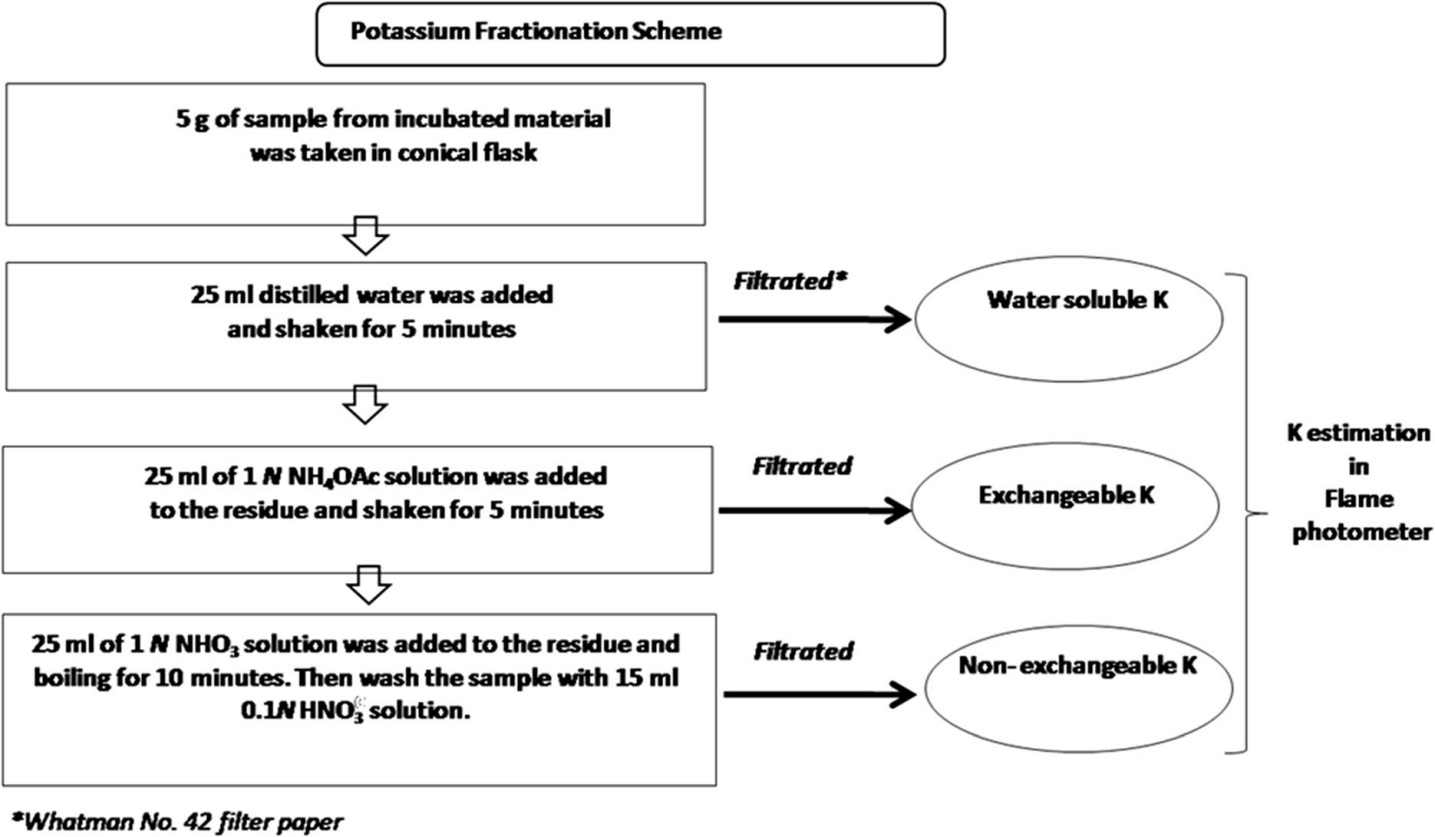
Flowchart depicting procedure of potassium fractionation scheme

The filtered leachates were also analyzed for Zn, Cu, Fe and Mn using ICP-MS (Agilent 7700 × ICP-MS, Agilent Technologies Inc., USA). The pH of the leachate was measured on a digital pH meter (PHM 93, Mettler Toledo, USA). At the end of the incubation, the mixture from each treatment was extracted with diethylenetriaminepentaacetic acid (DTPA) (Lindsay and Norvell 1978) and 0.05 M citric acid separately. The amounts of extracted Zn, Cu, Mn and Fe were measured on ICP-MS. The different fractions of K in each treatment were analyzed following the fractionation scheme depicted in Fig. 1. Water soluble, exchangeable and non-exchangeable K contents in the treatments were analyzed as per standard procedure (Page et al. 1982; Hanway and Heidel 1952; Wood and DeTurk 1940).

### Statistical analysis

Data generated from the study were represented as means of three independent experiments. The analysis of variance (one-way ANOVA) was conducted as per the experimental design (Completely Randomized Design). The critical difference at *p* =* 0.05* was estimated to compare differences between means of individual treatments. Microsoft Excel (Microsoft Corporation, USA) software packages were used for data calculation, tabulation and graphical representations.

## Results and discussion

### Characteristics of raw materials

The RMP was a by-product originating from mining activities of various minerals. The host rock mineralogy indicated that it was of volcanic origin with high N_2_O and K_2_O contents, and was formed by fractional crystallization of mafic parent magma (Jenkins and Nethery 1992). An earlier X-ray diffraction study of RMP indicated the presence of basic volcanic rock forming minerals (Basak et al. 2018). The RMP was predominated with quartz, anorthite, albite, some K-feldspar, mica and chlorite (Basak et al. 2018). The XRF data confirmed Si, Al, Fe, Mn and K contents in decreasing order, and smaller proportions of Cu, Ca, Mg and P in the RMP (Table 1). Owing to the nutritional contents, the RMP would have potential agricultural use, especially in nutrient-poor soils (Ramos et al. 2015, 2019). The PTE contents of RMP obtained through ICP-MS analysis indicated very low concentration of As (2.54 ppm), Cd (0.35 ppm), Hg (< 0.01 ppm) and Pb (0.59 ppm), which are not likely to pose an environmental risk (Dalmora et al. 2020a, b). A comparative chemical composition of the RMP and organic materials (CD and LS) are presented in Table 2. The RMP contained an appreciable amount of total K (1.54%) and micronutrients (14,754, 3098, 14,919 and 14,839 mg kg^−1^ Zn, Cu, Fe and Mn, respectively). Both the organic materials contained > 40% of total carbon. Apart from carbon, the LS contained notable amounts of N (1.93%) and K (1.13%), while CD contained 0.69% N and 0.11% K on dry weight basis.

**Table 1:** Chemical composition in percentage weight of oxide in the rock mineral powder

Oxides	Content (%)
SiO_2_	46.81
TiO_2_	1.24
Al_2_O_3_	37.61
Fe_2_O_3_	2.13
MnO	1.92
ZnO	1.82
CuO	0.33
MgO	0.59
CaO	1.25
Na_2_O	1.17
K_2_O	1.85
P_2_O_5_	0.04
SO_3_	1.29
LOI*	1.42
Total	99.47

*Loss on ignition

**Table 2:** Comparative chemical properties of the materials used in the study

Material used	pH	TC (%)	TKN	C: N ratio	TK (%)	Zn (mg kg^−1^)	Cu (mg kg^−1^)	Fe (mg kg^−1^)	Mn (mg kg^−1^)
Legume straw (LS)	5.4	42.3 (0.92)^*^	1.93 (0.17)	21.9	1.13 (0.09)	29.7 (1.44)	13.2 (0.79)	187.6 (2.92)	54.8 (3.24)
Cow dung (CD)	7.9	41.7 (1.12)	0.69 (0.10)	60.4	0.11 (0.007)	123 (9.2)	34 (2.8)	1121 (18.7)	266 (13.4)
Rock mineral powder (RMP)	8.7	–	–	–	1.54 (0.21)	14,754 (124)	3098 (207)	14,919 (14.7)	14,839 (234)

*TC* total carbon, *TKN* total Kjeldhal nitrogen, *TK* total potassium]

*Value in the parenthesis is standard deviation (SD), *n* = 3

### pH of leachate

**Fig. 2:**
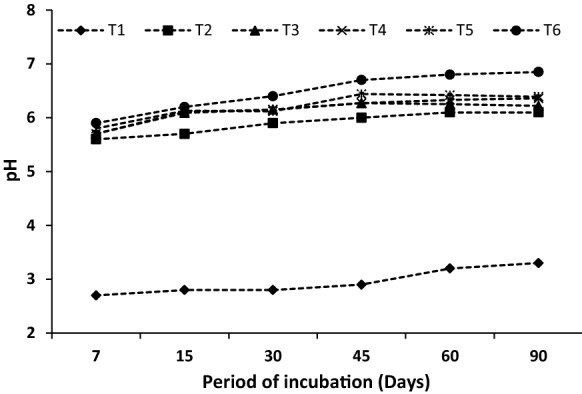
Effect of different treatments on the pH values of the collected leachate at different intervals of incubation time

In the case of quartz (control), the pH of the leachates was controlled by the pH of the eluent (i.e., 0.01 M citric acid), and the pH values varied from 2.7 to 3.3 irrespective of the incubation period (Fig. 2). However, treatments containing LS, CD and RMP resulted in notable changes of the leachate pH values during the incubation period. Treatments containing RMP showed significantly (*p* < 0.05) higher pH (5.7–6.8) in the leachate than the treatments without RMP (0.7 unit increase in pH at the upper end). There was an increasing trend in pH values with advancement of the incubation period, irrespective of the particle size of the RMP. Finer fraction of RMP had more pronounced effect on increasing the pH value than the coarser fractions. This might be due to faster dissolution of the finer fraction particles than coarser ones (Basak et al. 2018). The increase in pH with the introduction of LS and CD might be due to the mineralization of N-rich organic materials. Organic N contents in LS and CD were likely the main factor contributing to the pH rise of the mixture because the ammonification process during organic matter decomposition consumes H^+^ ions and raises the pH value (Wong and Swift 2003). The increase in pH with the addition of RMP might be explained by the acid neutralization capacity (ANC) of the mineral powder (Weber et al. 2005). The dissolution reaction involving silicate minerals would have the potential to neutralize acidity by consuming H^+^ ions (Eary and Williamson 2006). Mineralization of organic carbon leading to the production of OH^−^ ions by ligand exchange reaction as well as via the introduction of basic cations (Ca^2+^, Mg^2+^, K^+^ and Na^+^) during the incubation might have raised the pH of the medium (Anda et al. 2015). Increasing pH due to the positive liming effect from legume residue (Tang and Yu 1999) and RMP (Eary and Williamson 2006) was reported to ameliorate soil acidity to some extent. For example, olivine (Silva et al. 2013) and granite (van Noort et al. 2018) powders were used as an effective alternative to conventional liming materials for correcting acidity of agricultural and forest soils.

### Potassium release from RMP

**Fig. 3:**
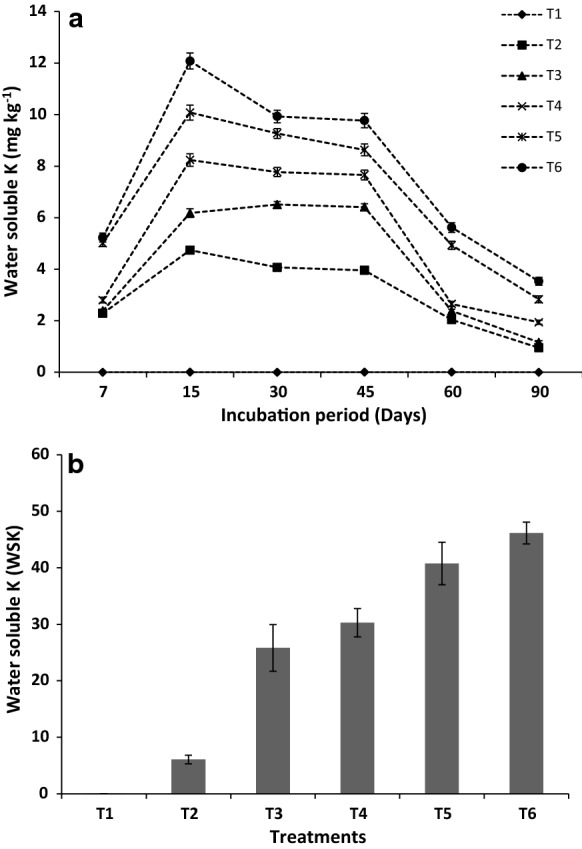
Water soluble K (mg kg^−1^ mixture) release patterns (**a**), and cumulative water soluble K release (**b**) from RMP during the incubation period

Water soluble K (WSK) released from various treatments during the leaching experiment is presented in Fig. 3. Results indicated that relatively higher amounts of K were released during the initial period of leaching from all the treatments than the control (*T*_1_). The K release rate sharply decreased up to 45 days of leaching and then gradually decreased up to 90 days. Overall, blending RMP with organic materials (LS and CD) showed significantly higher (2–3 folds) K release than organic material only treatment, irrespective of the leaching period. However, variation of K release among the treatments went down with the advancement of the leaching period. The treatment containing150 µm RMP (*T*_6_) accounted for the highest K release followed by *T*_5_ (250 µm), *T*_4_ (500 µm) and *T*_3_ (2000 µm). It might be due to the increase of reactive surface area with the increase of fineness of the particles. The intercept and slope values obtained from the first-order kinetic equation also indicated an increasing K release trend with finer RMP particles (Table 3). A higher dissolution rate can be expected as more exchange sites open up during the dissolution reaction (de Fatima Tavares et al. 2018). The current results agree with the findings of Harley and Gilkes (2000) and Basak et al. (2018) for K bearing silicate minerals.

**Table 3:** Rate constant (slope) and intercept of K release in leaching with water during incubation from four size fractions of rock mineral powder fitted into the first-order kinetic equation

Size fractions (µm)	First-order equation parameters	
	Slop × 10^−2^ (h^−1^)	Intercept (mg kg^−1^)
1000	7.23	1.42
500	7.55	1.53
250	7.83	1.66
125	8.09	1.89

The cumulative release of WSK from different treatments was computed by taking the sum of the amounts of WSK (mg kg^−1^) released at a particular time (Fig. 3). The cumulative WSK release was recorded notably higher in the case of treatment containing RMP as compared to the treatment containing only organic materials (*T*_2_). The smallest size fraction of the RMP (*T*_6_) recorded the highest cumulative WSK (46.2 mg kg^−1^) which was significantly higher than the largest size fraction (*T*_3_) of RMP (25.8 mg kg^−1^). Higher values of rate constant (slope) and intercept were obtained with smaller particle size of the RMP (Table 2). The K solubilization from minerals can be influenced by several geochemical factors, including pH, redox potential and chemical environment present in the plant rhizosphere (Zhu et al. 2014). The exact geochemical conditions of a natural environment might not be present under laboratory condition, but some factors such as pH and redox potential are expected to play important roles both under laboratory and field conditions (Wang et al. 2011). Current results indicated that organic materials improved the bioavailability of K from RMP during the incubation. Mobilization of K from the RMP with organic materials might have contributed considerably to WSK release (Basak et al. 2017). During incubation, the K release from the RMP through solubilization by organic acids produced from decomposition of organic matter might have attributed to WSK increase (Szmidt and Ferguson 2004). These results corroborated the outcome of other studies where a significant amount of K release from waste mica (Basak 2018) and phonolite (de Fatima Tavares et al. 2018) powders were studied when incubated with organic materials such as compost, cow manure and plant residues.

### Micronutrients release from RMP

Total release of micronutrients (Zn, Cu, Fe and Mn) in the leachate throughout the incubation period is presented in Table 4. A little amount of micronutrients was released from the treatment T_2_ containing only organic materials (LS and CD). The addition of RMP with organic materials recorded a significant (*p *<* 0.05*) increase of micronutrients release. The extent of micronutrients release increased with a decrease in particle size of the RMP, which followed the K release pattern as discussed earlier. About 18.7% Zn added as RMP was released over the incubation period. The Zn release increased from 4.7 to 23.2% with a decrease in the particle size of RMP from 2000 to 150 µm. The release of Cu varied from 2.9 to 21.6% of the total added amount via RMP, and a trend similar to Zn showing increased release with increasing fineness of particles was observed. The Fe and Mn release also followed the similar trend as in the case of Zn and Cu. Overall, Fe and Mn recovery from RMP recorded about 11.2 and 6.6%, respectively. These results indicated that micronutrients release during the incubation was significantly higher with the fine size fractions of RMP than the coarse particles.

**Table 4:** Total amounts of micronutrients (Zn, Cu, Fe and Mn) released from different treatments during the period (90 days) of incubation experiment

Treatments	Zn (mg)	Cu (mg)	Fe (mg)	Mn (mg)
*T*_1_: Control (quartz sand)	–	–	–	–
*T*_2_: Quartz + RMP	1.710	0.134	2.372	0.378
*T*_3_: Quartz + LS + CD + RMP (2000 µm)	1.883 (4.7)	0.251 (2.9)	2.510 (3.8)	0.526 (1.2)
*T*_4_: Quartz + LS + CD + RMP (500 µm)	2.962 (7.4)	0.906 (10.5)	5.330 (8.1)	1.623 (3.7)
*T*_5_: Quartz + LS + CD + RMP (250 µm)	6.765 (16.9)	1.535 (17.8)	9.015 (13.7)	3.642 (8.3)
*T*_6_: Quartz + LS + CD + RMP (150 µm)	9.287 (23.2)	1.864 (21.6)	12.632 (19.2)	5.792 (13.2)

The solubility of micronutrients depends on the pH and redox potential of the medium (Violante and Caporale 2015). The solubility of Zn is known to be more sensitive to pH, whereas Fe and Mn solubility notably depends on the redox potential (Pan et al. 2014). Additionally, the humic acid fraction of organic matter is responsible for immobilizing metal ions (Cu, and Zn) at pH < 6.5 (Chaudhary and Narwal 2005; Perelomov et al. 2018). On the other hand, Fe and Mn may convert to their reduced forms under an anaerobic (low redox) condition leading to higher Fe and Mn solubility. Under anaerobic condition, Fe^3+^ and Mn^4+^ act as electron acceptors, and are transformed to their reduced counterparts, Fe^2+^ and Mn^2+^ (Sahrawat 2005). Micronutrients speciation may approach in two opposite directions by organic matter application to soils. It can either reduce the availability by making insoluble metal complexes, or increase the availability by solubilization and release of organically complexed metals (Diacono and Montemurro 2010). In this study, experiments conducted under controlled conditions were conducive for decomposition of the organic materials, and an optimum microbial activity could be expected. It was reported that most of the released micronutrients from organic matter might get re-fixed in the soil matrix (Stevenson 1991), which was not expected here as the experiment was conducted without a soil as such. However, a decrease in micronutrient content was likely due to the formation of water insoluble metal complexes (Chaudhary and Narwal 2005). In the current experiment, exact soil conditions were not possible to create; but a weak citric acid was added to make the environment quite similar to the rhizosphere. As a result, the micronutrient release in the leaching experiment did not follow the trend that would exactly happen in the soil environment. Nevertheless, it was possible to give an estimate of the micronutrient availability from RMP for plant nutrition by simulating the plant rhizosphere chemistry using citric acid (Wang et al. 2015). Results of the present study thus showed that RMP with organic materials could serve as a potential source of Zn, Cu, Fe and Mn in soils.

### Potassium fractions after incubation

Different fractions of K, i.e., water soluble K (WSK), exchangeable K (Ex-K) and non-exchangeable K (NEx-K), in RMP were significantly influenced during the incubation and leaching experiments (Table 5). The amount of WSK was recorded less as compared to Ex-K and NEx-K. A substantial amount of WSK was released during the successive leaching events, which might have caused the changes in K fractions. The treatment containing only organic materials (*T*_2_) recorded a significant amount of WSK (0.94 mg kg^−1^), but contained a negligible amount of Ex-K and NEx-K (Table 5). There was an increasing trend of K pools (WSK, Ex-K and NEx-K) with decreasing particle size of the RMP. The treatment containing the smallest particle size (*T*_6_) recorded significantly (*p *<0.05) higher Ex-K (28.2 mg kg^−1^) and NEx-K (272.8 mg kg^−1^) than WSK (2.87 mg kg^−1^). The readily dissolvable K present on the surface of RMP particles was first removed by distilled water (WSK). Then, dilute salt solution (1 N NH_4_OAc) able to extract K by exchanging with cations at the surface and wedge zones of the particles was considered as Ex-K. The low-molecular weight organic acids such as citric acid having functional groups (–OH and –COOH) tend to form organo-metal complexes with metal ions present in the mineral structure (Lian et al. 2002), thus accelerating the K release from RMP. A large amount of K was extracted by HNO_3_ from the mineral structure (*T*_6_) due to the release of K from partially opened and inter-layer positions of minerals (Moritsuka et al. 2004). Overall, the high K release from the fine particle size fraction of the RMP was likely to be due to the greater reactive surface area relative to that of the coarser particles (Priyono and Gilkes 2008).

**Table 5:** Effect of various treatments on different fractions of K after completion of the incubation

Treatments	WSK	Ex K	Non Ex K
	(mg kg^−1^ mixture)		
*T*_1_: Control (quartz sand)	0.00	0.00	0.00
*T*_2_: Quartz + RMP	0.94	15.6	84.3
*T*_3_: Quartz + LS + CD + RMP (2000 µm)	1.16	16.8	86.7
*T*_4_: Quartz + LS + CD + RMP (500 µm)	1.94	19.6	120.5
*T*_5_: Quartz + LS + CD + RMP (250 µm)	2.53	20.5	178.3
*T*_6_: Quartz + LS + CD + RMP (150 µm)	2.87	28.2	272.8
CD*(*p* = 0.05)	0.17	2.87	7.82

*WSK* water soluble K, *Ex K* exchangeable K, *Non Ex K* non-exchangeable K

*Critical difference

### DTPA and citric acid extractable micronutrients after incubation

DTPA and citric acid are considered to be suitable extractants for studying plant available micronutrients in soils (Bibiso et al. 2016). Both the extractants act as organic ligands; they reduce the activity of micronutrient cations in the solution through formation of soluble organo-metal complexes. Both the methods are well correlated for extracting plant available micronutrient cations from the soil (Samourgiannidis and Matsi 2013). Micronutrients extracted by DTPA and citric acid at the end of the incubation are presented in Table 6. Micronutrients release by both the extractants were significantly (*p *<0.05) higher in the treatments receiving RMP than the control. Here again for both the extractants, micronutrients release increased with a decrease in particle size of the RMP. The highest amount of Zn (7.34 mg kg^−1^) was extracted with citric acid in the treatment *T*_6_ containing quartz + LS + CD + RMP (150 µm), which was significantly (*p *<0.05) higher than the rest of the treatments (Table 6). A similar trend was observed in the case of micronutrients extraction by DTPA. There were significant differences (*p *<0.05) in releasing Fe and Mn due to a decrease in particle size of RMP, either extracted with DTPA or citric acid. Under both DTPA and citric acid extractions, the highest Fe (8.84 mg kg^−1^) and Mn (7.54 mg kg^−1^) release was recorded with treatment *T*_6_ receiving the finest RMP particles. On average, increased micronutrients (Zn, Fe and Mn) release was observed with citric acid than DTPA. This difference was higher in case of fine sized RMP particles than large particles. Citric acid has high chelating potential to trap metal cations due to its tridentate structure, and thus can effectively displace adsorbed anions from mineral surfaces (Wang et al. 2015). Various mechanisms including ligand-exchange reaction, dissolution of metal oxides from surfaces and formation of metal–organic complexes attributed to higher micronutrient cations extraction by citric acid than DTPA (Shan et al. 2002). The release of Cu from various treatments followed different trend than that of other micronutrients. Notably lower Cu release was observed irrespective of treatments and extractants than other micronutrients (Zn, Fe and Mn). Copper forms inner sphere complexes consisting of one to two five-membered chelate rings in association with functional groups of organic matter (Diaz-Barrientos et al. 2003). This suggested that Cu was strongly bound with organic matter functional groups within the study pH range, and subsequently its availability could be reduced in the environment (Maqueda et al. 2015). The Cu release was lower with citric acid compared to DTPA but not showing a significant difference among the treatments, which warrants further investigation.

**Table 6:** Effect of different treatments on extractable micronutrient (Zn, Cu, Fe and Mn) contents with DTPA and citric acid after completion of the incubation

Treatments	Zn	Cu	Fe	Mn
	(mg kg^−1^ mixture)			
DTPA				
*T*_1_: Control (quartz sand)	0.00	0.00	0.00	0.00
*T*_2_: Quartz + RMP	1.21	0.24	3.24	3.43
*T*_3_: Quartz + LS + CD + RMP (2000 µm)	2.57	0.33	4.83	4.12
*T*_4_: Quartz + LS + CD + RMP (500 µm)	2.79	0.47	5.26	4.78
*T*_5_: Quartz + LS + CD + RMP (250 µm)	3.83	0.54	6.40	5.23
*T*_6_: Quartz + LS + CD + RMP (150 µm)	5.32	0.67	7.37	6.87
CD* (*p* = 0.05)	1.22	0.13	2.66	1.57
Citric acid				
*T*_1_: Control (quartz sand)	0.00	0.00	0.00	0.00
*T*_2_: Quartz + RMP	2.43	0.09	4.23	3.28
*T*_3_: Quartz + LS + CD + RMP (2000 µm)	3.54	0.13	5.42	5.12
*T*_4_: Quartz + LS + CD + RMP (500 µm)	4.17	0.19	6.31	5.56
*T*_5_: Quartz + LS + CD + RMP (250 µm)	5.66	0.27	7.92	6.09
*T*_6_: Quartz + LS + CD + RMP (150 µm)	7.34	0.34	8.84	7.54
CD* (*p* = 0.05)	1.09	0.10	2.43	0.72

*Critical difference

Results of the present investigation indicated that Zn, Fe and Mn extracted from RMP at the end of the incubation by 0.05 M citric acid and DTPA followed similar patterns except for Cu. DTPA is considered to be an ideal extractant to study plant available micronutrients in the soil (Bibiso et al. 2016), and can be used as a standard reference to test the efficiency of other extraction methods. The DTPA method was originally developed for calcareous soil. However, results from the present study indicated that the method was equally effective for the estimation of micronutrients in an acidic condition. Micronutrients extraction by 0.05 M citric acid here followed a similar pattern as DTPA, but quite higher values for Zn, Fe and Mn were obtained (Table 5). However, citric acid did not show the increase of Cu extraction in the same way as DTPA method did. This may be explained by the reduction of Cu availability due to the formation of strong complex with functional groups of organic matter (Karlsson et al. 2006), as described earlier. Results indicated that the RMP blended with organic materials could be used to meet Zn, Cu, Fe and Mn requirements of crops in organic agriculture. These results agree with previous reports where the application of silicate minerals improved growth and yield of maize (Basak et al. 2018), wheat (Bolland and Baker 2000), sorghum (Kelland et al. 2020), and sugarcane (Theodoro and Leonardos 2006). The addition of RMP with organics showed a synergistic effect on micronutrients release in adequate amounts to meet crop needs (Garcia-Mina et al. 2004).

### Environmental and agricultural implications

This study was conducted under controlled laboratory conditions. An important question remains open whether the release of K and micronutrients from RMP was sufficient to meet the demand of agricultural crops in sustainable production. A pot culture study conducted earlier showed significant improvement of yield, K and micronutrients uptakes in plants (*Zea maize* and *Ocimum sanctum*) treated with the same RMP (7.5% application rate) collected from Seaham quarry, New South Wales, Australia (Basak et al. 2018). Another important concern is environmental risks associated with the potential release of harmful elements from RMP. The elemental compositions of different RMP studied in majority of the studies did not show any environmental or human health risk (van Straaten 2006; Kelland et al. 2020). However, waste minerals obtained from sludge was found to contain critical amounts of Pb, As and Cr, and not allowed to use in agriculture (Madaras et al. 2013). On the other hand, rock dusts collected from geological deposits and mountain sides were evaluated under agricultural regulations, and were found to be free of toxic elements (Manning 2018). The RMP used in the present investigation was evaluated earlier in a pot study, and did not show any PTE risk (Basak et al. 2018). RMP release nutrient elements slowly over the course of weathering and continuously supply them to plants throughout the plant growth period (Harley and Gilkes 2000). There is negligible risk of over-dosage of a single nutrient with RMP unlike often seen in the case of soluble fertilizers, and the slow weathering makes RMP less prone to off-site contamination too (Gillman et al. 2002).

In the current study, organic materials (cow dung and legume straw) and citric acid were used to simulate a soil chemical environment quite similar to a rhizosphere soil (Violante and Caporale 2015). A normal yield (4000 kg ha^−1^) of maize (90–100 days) removes 372 g Zn, 3648 g Fe, 780 g Mn and 308 g Cu ha^−1^ from an agricultural soil (Takkar 1996). Considering the release of Fe during the incubation period (90 days), 165.5 kg RMP ha^−1^ is required to supplement the amount removed by a maize crop. This amount of RMP can supply variable amounts of K and other micronutrients, i.e., smaller or greater than the crop demands due to variable contents of K and micronutrients in the RMP (Table 2) and their variable release patterns (Table 4). In this theoretical calculation, K and micronutrients supply from the native soil is not included, which may underestimate the real-time effect of RMP on crop yield. Nevertheless, this study demonstrated the potential of RMP as K and micronutrient sources, and intrigued to conduct further field level evaluation of the RMP combined with organic matters under ecological farming systems.

## Conclusions

Results of this study suggested that the RMP contained significant amounts of K (1.54% total K) and supplied essential micronutrients (Zn, Cu, Fe and Mn) when used in combination with organic materials. During the incubation, 23.2, 21.6, 19.2 and 13.2% of total Zn, Cu, Fe and Mn present in RMP were released. The RMP thus could be considered as a potential source of K as well as micronutrients in crop production without any PTE accumulation risk. The combination of organic materials and RMP in soil fertility replenishment strategies should further be investigated. Plant roots can accelerate weathering of minerals through the prevalence of microbial activity hotspots, production of organic acids, and a low pH environment. Plant intervention studies should be undertaken to test the potential use of the RMP in organic farming. An effective blending of RMP with organic amendments could be a potential source of K and micronutrients in the organic farming system.

## Data Availability

The data supporting the findings of this study are available within the article [and/or] its supplementary materials.
